# Discovery of Crystallized and Weakly Coupled Aggregates of Pseudocyanine Iodide

**DOI:** 10.1021/acs.jpcb.5c06896

**Published:** 2026-01-21

**Authors:** Autumn R. Bruncz, Arka Chatterjee, Henry Gatica-Gutierrez, Sadie Brasel, Alexey Belyanin, Anna-Karin Gustavsson, Shengxi Huang

**Affiliations:** † Department of Electrical and Computer Engineering, 3990 Rice University, Houston, Texas 77005, United States; ‡ Department of Chemistry, Rice University, Houston, Texas 77005, United States; § Department of Physics & Astronomy, 14736 Texas A&M University, College Station, Texas 77840, United States; ∥ Department of BioSciences, Rice University, Houston, Texas 77005 United States; ⊥ Smalley-Curl Institute, Rice University, Houston, Texas 77005 United States; # Center for Nanoscale Imaging Sciences, Rice University, Houston, Texas 77005, United States; ∇ Department of Cancer Biology, University of Texas MD Anderson Cancer Center, Houston, Texas 77005 United States

## Abstract

We present a new class of pseudocyanine iodide (PIC-I) aggregates formed by freeze-induced self-assembly into layered ribbon structures. Unlike conventional PIC J-aggregates with head-to-tail dipole alignment, these ribbons adopt a complex and mixed HJ-aggregate arrangement. Unexpectedly, the aggregate ribbons exhibit intense, red-shifted fluorescence, in contrast to the typical nonemissive nature of their monomer form. At 4 K, their emission lifetimes range from ∼300 ps to ∼1 ns, substantially longer than those of J-aggregates. The combination of red-shifted emission, monomer-like absorption, and extended lifetimes reveals their mixed packing contributions. Second-order autocorrelation measurements with a Hanbury Brown and Twiss interferometer show photon bunching, providing evidence for cooperative emission from collective excitonic states—an effect not previously observed in any aggregates larger than dimers. These findings establish PIC-I HJ-aggregate ribbons as a unique platform for exploring collective photophysics in mixed aggregate molecular assemblies, suggesting potential applications in bioimaging, light-emitting devices, and sensing.

## Graphical abstract

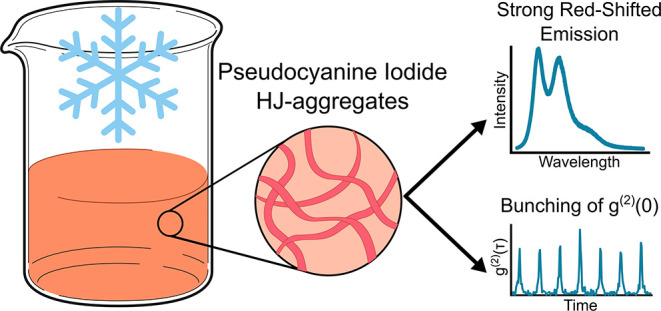

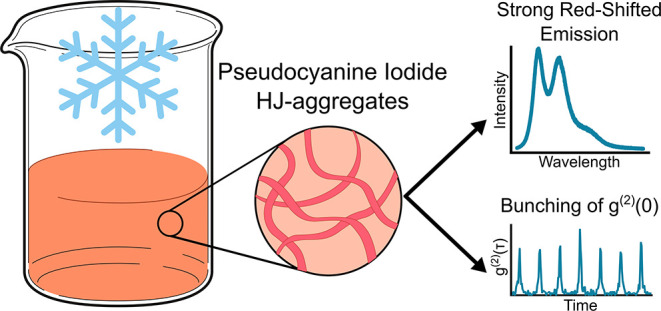

## Introduction

Since their discovery by Jelley and Scheibe in the 1930s, molecular aggregates have intrigued scientists due to their shifted absorption spectra and either enhanced or quenched fluorescence.^1–4^ According to Kasha’s theory (1960s), the orientation and coupling of transition dipole moments (TDMs) within molecular aggregates lead to shifts in excited-state energies.^5^ The orientation between the molecules determines the aggregate type and its emission properties.^6,7^ J-aggregates, so named for Jelley, display a narrow red-shifted absorption band compared to monomer absorption, referred to as the J-band. J-aggregates exhibit enhanced emission compared to their monomer form, with a small Stokes shift from their J-band.^8–11^ For J-aggregates, Kasha’s theory describes their TDMs as head-to-tail. Their H-aggregate counterparts display a blue-shifted broad absorption peak, with suppressed or quenched fluorescence.^7,12–14^ In contrast to the head-to-tail alignment in J-aggregates, H-aggregates have a side-by-side TDM orientation.^7^ Molecular aggregate arrangements can range from simple linear chains of molecules to large and complex 2D or 3D structures with varied orientations, resulting in long-range and short-range coupling. Additionally, charge transfer and vibronic coupling between molecules can affect the optical characteristics of an aggregate.^6,7,15–19^ These properties help explain complex aggregate behavior like the formation of HJ-aggregates, which have both J- and H-aggregate properties that can be thermally turned on or off,^7,20–22^ or null aggregates, where their coupling would essentially cancel out to result in properties the same as their monomers.^7,20^

One of the most well-studied of these aggregating molecules is pseudocyanine (PIC),^23–32,32–35^ employed by Jelley and Scheibe in their initial reports on J-aggregates.^1^ PIC easily forms J-aggregates in aqueous solution and is commercially accessible, making it a popular choice for studying aggregate behavior.^8,9,25^ PIC J-aggregates typically form nanoscale fibers, roughly 2–3 nm in width as revealed by cryoTEM, and have been theorized to form a brickwork molecular packing arrangement.^23,36^ Most commonly, PIC exists in an isomorphous form with a counterion of iodide (PIC-I), chloride (PIC-Cl), or bromide (PIC-Br).^35^ The formation of J-aggregates of PIC is highly dependent on temperature and dye concentration, resulting in J-aggregate phase diagrams.^37^ In addition to the studies performed on PIC J-aggregates, scientists have been able to study PIC’s H-aggregate form. PIC H-aggregates have been shown to form with the aid of a surfactant,^38^ as dimers or trimers in PIC solutions,^37,39^ or through nanotube scaffolds.^40^

To potentially form new aggregate arrangements, we developed a method by freezing a PIC-I solution, which is more likely to form J-aggregates at low temperatures.^37^ The synthesized aggregate assumes a ribbon structure with a width range of 0.9–6.6 μm. We found the ensemble absorption of the ribbons to be similar to that of the monomer, but with peak ratios closer to those of J-aggregates. Additionally, their emission was red-shifted compared to the monomers and J-aggregates and resembled a vibronic progression. The estimated average quantum yield (QY) increased to ∼8% compared to its monomer forms of 0.04%,^41^ suggesting the formation of an aggregate with mixed coupling contributions, resulting in weak coupling overall. Polarization-dependent studies revealed anisotropic behavior toward the short axis of the ribbons, implying that the frozen molecules form an ordered structure. Fluorescent lifetimes of the J-aggregates were short at 4 K, ∼200 ps, and remained relatively constant at room temperatures. Conversely, the ribbon structures had longer lifetimes of ∼700 ps at 4 K, which then decreased to ∼200 ps at room temperature due to exciton–phonon coupling and the onset of J-like coupling. These results helped confirm that HJ-aggregate packing most likely exists in the frozen 3D structure. Lastly, we found photon bunching, a signature of cooperative emission, at some locations on the ribbon aggregate sample. This result was not previously observed on J-aggregates or any aggregate structure larger than two molecules.^42^ Our work presents a practical synthesis method for previously unstudied PIC-I HJ-aggregates and provides valuable insight into their optical properties, expanding the knowledge of weak overall coupling in PIC-I and lending useful tools for future development of mixed coupling designer aggregates.

## Materials and Methods

Pseudocyanine iodide (1,1′-Diethyl-2,2′-cyanine iodide) was purchased from Sigma-Aldrich in powder form without further purification. PIC stock was formed by diluting the powder in methanol to a 10 mM concentration. The PIC stock was diluted in 0.2 M NaCl aqueous solution and sonicated for 20 min to form J-aggregate solutions. After sonication, the solution was heated at 80 °C for 30 min, allowing the methanol to boil off and leaving the final concentration of the solution to be 1 mM of PIC-I, unless expressed otherwise. This approach has previously been reported as a standard protocol for preparing PIC-I J-aggregates.^24,32,34^ For the development of HJ-aggregates, the J-aggregate solution was cooled to room temperature and then placed in a freezer (at 0 °C) for at least 24 h, inducing the HJ-aggregates to form. In contrast, the solution could be left as a liquid for J-aggregate formation. Experiments on PIC monomers were performed with PIC-I dissolved in methanol.

To prepare samples for measurements, the HJ-aggregate frozen solution was removed from the freezer and defrosted until it had just reached a liquid form (∼15 °C). Both HJ- and J-aggregates were drop-cast onto a substrate and dried with pressurized air. A sugar solution was then drop-cast onto the sample and left to dry in a desiccator in the dark for 3 h. The sugar solution, a 50:50 ratio of sucrose and trehalose by weight, was dissolved in Milli-Q water and formed a clear glassy matrix that stabilizes the aggregates and prevents possible deformation from the environment.^43–45^ HJ-aggregates did not dissociate while the sugar layer was drying due to the thin layer as well as the sucrose/trehalose slowing water dynamics.^46^ All PIC-I solutions were kept dark during sample preparation to prevent photobleaching of the molecules.

Temperature-dependent experiments were performed with the Montana Instruments Cryostation CryoAdvance-50. The Cryostation utilizes a helium compressor to reach temperatures as low as 3.5 K.

Photoluminescence (PL) measurements were performed with a Renishaw inVia confocal microscope equipped with a CCD camera. Spectra were collected using a 100× objective with 0.9 NA for room temperature and temperature-dependent measurements. Emission and excitation polarization measurements were performed using a linear polarizer and a half-wave plate in the optical collection and excitation path.

Time-resolved PL and second-order autocorrelation measurements were carried out using a PicoQuant MicroTime 100 upright time-resolved photoluminescence microscope. The excitation source was a diode laser emitting at 509 nm, which could be operated either in pulsed or CW mode. The laser was used in pulsed mode with a repetition frequency of 40 MHz and a 96 ps pulse width. The laser was directed to a high numerical aperture (100×, NA = 0.9) objective lens for room temperature measurements. For variable temperature measurements using the Cryostation, a 50×, NA = 0.35, long working length objective was used. Imaging and positioning of the laser spots were carried out via objective scanning using a 3D piezo scanner. The detection light was filtered with a 532 nm dichroic mirror, followed by a long pass 550 nm filter, before coupling into a graded-index multimode fiber (0.22 NA) with a fiber aperture of 62.5 μm acting as a confocal pinhole. A flip mirror directed the emitted light to either a spectrometer (FluoTime 300, PicoQuant) or two avalanche photodiodes (Excelitas Technologies) in a Hanbury Brown Twiss configuration for the autocorrelation measurements. A 50:50 beam splitter was used to divide the incoming photons, followed by a 590 nm bandpass filter with a fwhm of 10 nm placed before each of the detectors. Lifetime measurements and steady state PL for QY determination were carried out in the FluoTime 300 spectrometer equipped with a PMA-Hybrid 50 detector.

Ensemble absorption measurements were taken with the Thermo Scientific Invitrogen Nanodrop. Two μL of PIC-I solution was placed on the pedestal and cleaned with 70% ethanol and a Kimwipe after each measurement. The spectrophotometer’s autoranging path length from 0.03 to 1 mm allowed for measurements to be performed on highly concentrated samples of PIC-I, which made traditional spectrometers unable to register their transmittance. We measured the absorption in an ensemble in solution to allow dyes to remain highly concentrated, as is required for most self-assembled aggregates.

Steady-state PL spectra were fitted with Gaussian fits. The HJ-aggregate data was fitted with three peaks, while the J-aggregate peaks were fitted with two. Fittings were carried out on MATLAB with a nonlinear regression model. Ensemble absorption spectra were fitted the same way as steady-state PL. The fitting of the fluorescent lifetimes was carried out with Picoquant’s EasyTau 2 software. An iterative reconvolution using the instrument response function (IRF) of the system was used to fit a single exponential to the decays. SEM was performed using Apreo 2 SEM.

## Results and Discussion

As discussed in the methods section, PIC-I HJ-aggregates were achieved by freezing J-aggregate solutions. Optical microscopic images revealed large fiber-like structures, as seen in Figure 1A. These structures were significantly larger than the small fibrous PIC-I J-aggregates, which cannot be observed with optical microscopy.^23,36^ Microscopic images also revealed that the long structures have multiple colors with jagged or pointed ends. The ribbons’ orientation is random, and they are dispersed on the substrate, not clumping together often. We further analyzed the distribution of length and width of these ribbons out of 4 microscopic images taken of 1024 structures. The range of width, length, and aspect ratio was 0.9–6.6 μm, 4.6–89.4 μm, and 2.2–40.1, respectively. These results indicate a wide variation in the length and aspect ratio of the ribbons formed, while their width stays in a general range. More detailed results of the image analysis can be found in SI Figures S1 and S2. The largest ribbon recorded had a width of ∼10 μm and a length over 1 mm, shown in Figure S3. SEM images revealed that the structures take the form of ribbons, as seen in Figure 1B (additional SEM images in Figure S4). Some ribbons, like in Figure 1B, displayed a twist in their structure. We additionally performed atomic force microscopy (AFM) on the aggregates to reveal a ribbon structure with a twist, as shown in Figure 1C. A typical ribbon shows a width of 1.5 μm and a height of 0.6 μm, where twists can exist in the ribbon.

**Figure 1:**
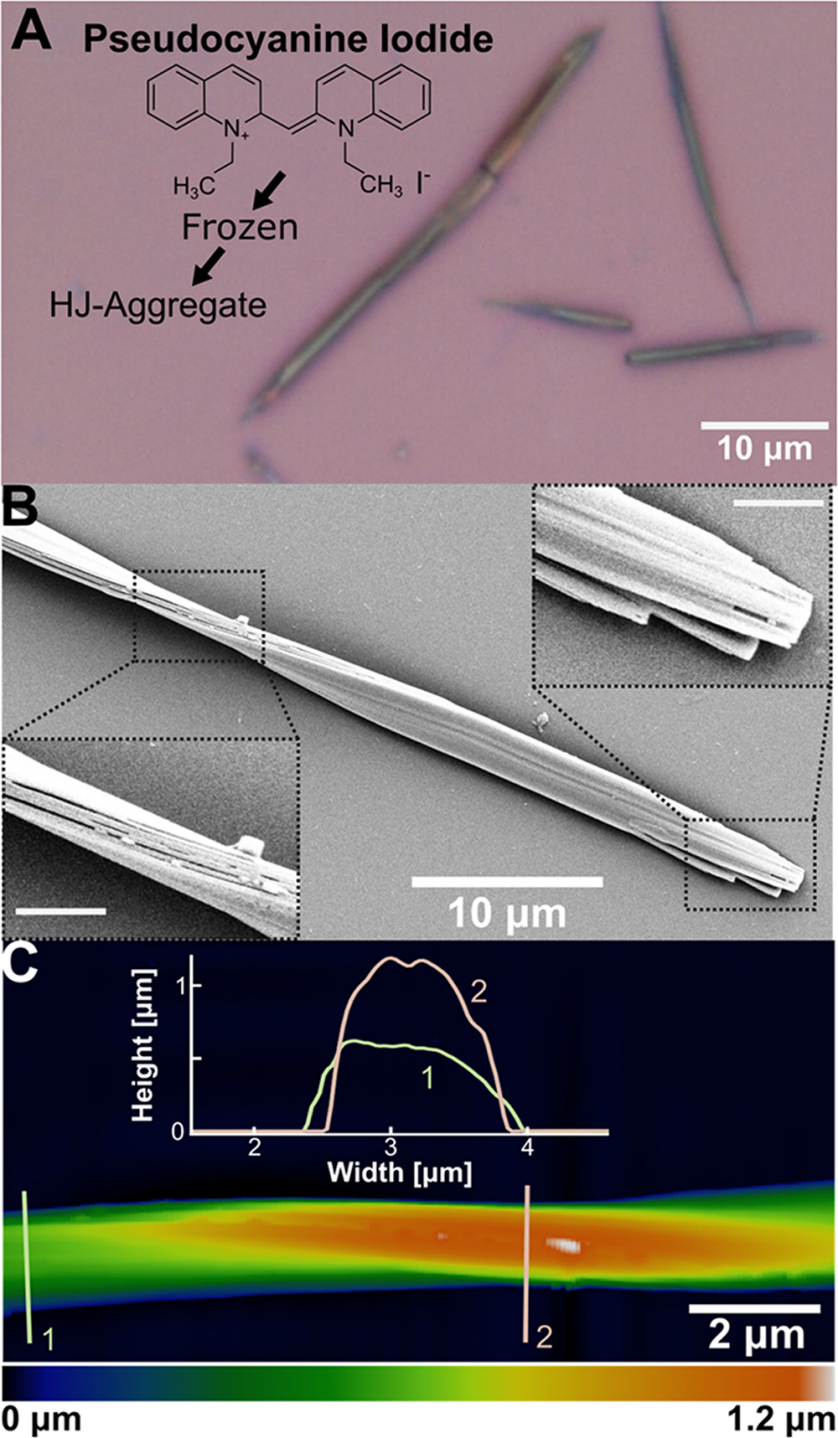
Structure and images of PIC-I HJ-aggregate nanoribbons. (a) Optical microscope image showing the chemical structure of HJ-aggregates; (b) SEM image of a single nanoribbon with insets of a twisted region and termini (scale bars = 2 μm); (c) AFM image of a twisted region with inset height profiles along lines 1 and 2.

The PIC J-aggregate structure has been well described as small fibers with a width of ∼2 to 3 nm, almost 3 orders of magnitude smaller in width than the large ribbons found in our work.^23,36^ This ribbon aggregate structure has never been reported in PIC-I. Another large aggregate structure of PIC-I has been recently reported by Zhu et al.^47^ However, their structure was formed by slowly bringing their solution to room temperature, which created a hexagonal nanowire that their authors characterize as an H-aggregate.^47^ J-aggregates of PIC-I have been recorded to form more readily at low temperatures, indicating that the J-aggregate growth through chain polymerization is kinetically favorable in colder liquid environments.^37^ At warmer temperatures, only the monomer and small oligomers of H-aggregates exist in solution. However, by freezing the solution, we hypothesize that the J-aggregates are forced together into pockets of high concentration, which then arrange into the new kinetically favorable ribbon shape. We suppose that this packing introduces H-like coupling, leading to complex and mixed coupling optical properties, which we later characterize as HJ-aggregates. By depositing the solution at different temperatures after being defrosted, we find that the occurrence of ribbons, especially large ribbon structures, greatly decreases, with only a few ribbon structures observed once the solution reached room temperature, as displayed in Figure S5. This occurrence of larger ribbon structures at lower temperatures, which then dissipate at room temperatures, supports our theory of cryoconcentration. It should be pointed out that the exact structure of PIC-I J-aggregates is still debated. While originally many proposed a general brickwork model,^48^ some other works have explored a rod-like chain,^36^ helical structure,^23^ or zigzag structure.^49^ It has been suggested that there are many packing orders and orientations of the TDMs of PIC that will change depending on the concentration, environment, or substrate of the aggregates.^9^ With the exact structure of PIC-I aggregates being elusive, future work will also be required to try to quantify the exact microscopic structure of these new HJ-aggregates, which we suggest form from the close contact of pre-existing J-aggregates. Additionally, we have not experimented on other derivatives of PIC, most notably, PIC-Cl and PIC-Br. We hypothesize that other forms of the dye will not form ribbon structures, or it will be more difficult. Reports have claimed that larger counteranions will more readily form H-aggregates.^50,51^ With iodide being the largest anion of the three most common counteranions, we hypothesize that the iodide allows for the H-like coupling that results in the weak coupling of our HJ-aggregates.

We determined the PIC-I ribbons as HJ-aggregates through spectral characterization. We completed liquid ensemble absorption measurements of the PIC-I ribbons, J-aggregates, and monomers, as shown in Figure 2A. Sample preparation for this measurement is described in the methods section. The monomer form of PIC-I has three main peaks that have been well-established in literature, with the peaks at 523 nm (A_1_), 486 nm (A_2_), and the sideband at 457 nm (A_3_) assigned as a typical vibronic progression of PIC-I.^52–54^ These peaks were present in the J-aggregate spectra, but with a lower intensity, suggesting that PIC-I monomers exist within the J-aggregate solution. The J-aggregates display a narrow peak at 573 nm, called the J-band, which aligns well with previous reports.^25,36^ The relatively low intensity of the J-band peak is most likely due to the low concentration of PIC-I used here for all of the samples, ∼0.5 mM. The HJ-aggregates or ribbons were difficult to measure due to their energetic unfavorability in liquid form. To prevent their dissociation back to J-aggregates, the HJ-aggregate samples were kept in dry ice, and their experiments were performed immediately after defrosting (approximately 288 K). To ensure the most accurate absorption spectrum was recorded, the measurements were performed 5 times and averaged, with the solution allowed to sit in a dry ice cooler for 15 min between each scan to maintain temperature. For the HJ-aggregate solution, there is the presence of a slight J-band due to some J-aggregates in the HJ-aggregate solution. The ribbon absorption remained very similar to its monomer counterpart but had a lower intensity than the monomer at a similar concentration, indicating the presence of aggregates. The lack of J-band or blue-shifted peaks below 500 nm indicates that these are not ideal H- or J-aggregates.^40,55,56^ To elucidate the absorption properties, we plot the ratio of the A_1_/A_2_ monomer peaks for the PIC-I monomer, J, and HJ-aggregates at varying concentrations, as shown in the inset of Figure 2A. The ratio is large for the PIC-I monomer but starts to decrease at higher concentrations due to the dipole coupling from the molecules’ forced proximity. The PIC-I J-aggregates A_1_/A_2_ ratio is smaller than that of the monomers and decreases more rapidly than the monomers with increasing concentration. This rapid decrease in the ratio suggests a higher aggregate presence at high concentrations, as previous reports indicate. Notably, the HJ-aggregates have a similar A_1_/A_2_ ratio to the J-aggregates, which rapidly decreases, but then reaches a threshold. For H-aggregates of PIC, which have been previously studied in dimers and trimers, their absorption ratio would be expected to be below 1, with the A_2_ peak dominating their absorption line shape.^37^ The similarity of the A_1_/A_2_ ratio implies the existence of an aggregate species of PIC-I, but does not clearly express itself as an ordered J- or H-aggregate. This result is consistent with previous theoretical and experimental works on null and HJ-aggregates. Due to the complex molecule packing, as well as charge transfer, these aggregates display absorption line shapes very similar to their monomer counterparts, but with changing peak intensity ratios.^7,20^ We additionally performed absorption measurements on the HJ-aggregate solution every 4 s for 36 s. We found that the J-band significantly grew before saturating after ∼30 s, as seen in Figure S6. This result, along with the diminished occurrence of ribbons when the solution is at higher temperatures, as discussed earlier, supports our claim that the HJ-aggregates are formed from J-aggregates. It could also be that the dissociation of the HJ-aggregates and the self-assembly into J-aggregates occurs so quickly in liquid that the HJ-aggregate features are lost before we can observe them. This result indicated that the ribbons would need to be kinetically trapped by drop-casting or spin-coating to accurately measure their spectral properties.

**Figure 2:**
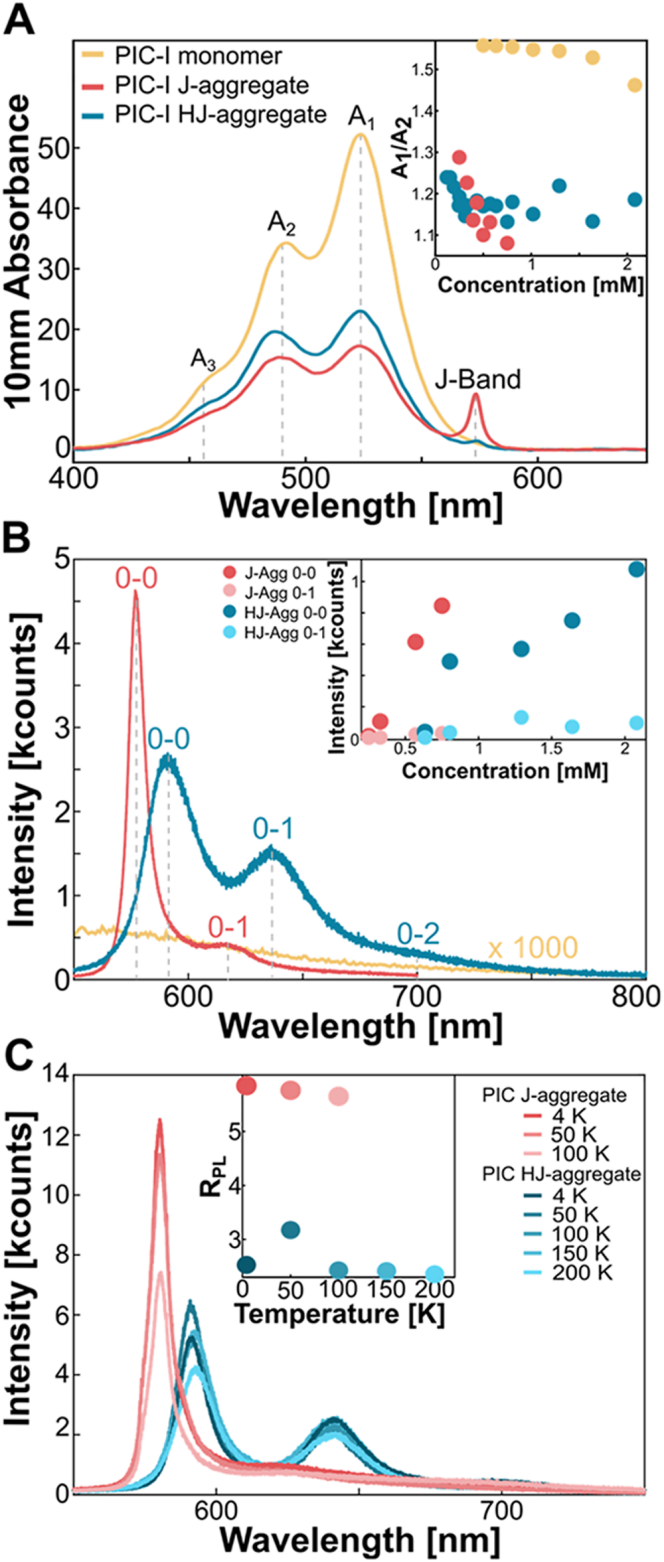
Steady-state spectral characteristics of PIC-I monomer and aggregates. (A) Ensemble absorption spectra of J-aggregates (red), HJ-aggregates (blue), and monomer (yellow) in liquid, with inset showing ratio of A_1_/A_2_ peaks at varying concentrations (B) PL spectra of J-aggregates, HJ-aggregates, and monomer excited at 532 nm and measured at 295 K. Monomer emission multiplied by 1000 to be seen in spectra. Inset: Intensity of 0–0 (dark) and 0–1 (light) transition peaks for J- (red) and HJ-aggregates (blue) (C) Temperature-dependent PL spectra of J- and HJ-aggregates, with inset showing *R*_PL_ at different temperatures (paler colors indicate higher temperatures).

To further corroborate the results from ensemble liquid absorption measurements, we performed microscopic photoluminescence (PL) experiments of drop-cast individual structures, shown in Figure 2B. The beam spot with a 100× objective was diffraction-limited to 300 nm, much smaller than the average width of the ribbon, allowing us to focus entirely on the ribbon structure. The monomer of PIC-I exhibits quenched fluorescence, with low QY, and vibronic progression with peak wavelength at approximately 550 nm.^24,35^ The narrow, red-shifted emission of the J-aggregates centered at 580 nm with a vibronic shoulder at 620 nm also aligns well with prior reports.^23,25,26,35,57^ The HJ-aggregates have a notable emission, with three peaks: two at 590 and 640 nm and a shoulder at 700 nm, mirroring the vibronic progression of the absorption, but further red-shifted than the monomer and J-aggregates. The average QY of these ribbon aggregates is estimated to be ∼8%, details of which are displayed in Figure S7. This value is an order of magnitude smaller than the J-aggregates, which have been reported to be as high as 44%,^58^ but much larger than the monomer QY of 0.04%.^41^ This red-shifted and stronger emission than the monomer gives evidence that some aggregation event is occurring for the PIC-I molecules. The QY result could imply aggregate induced emission (AIE) or a quenched nonradiative decay pathway resulting from disordered alignments of molecules.^59^ However, due to the molecule’s precise ordering into J-aggregates at low temperatures, as well as later experimental results, we conclude that an ordered aggregation resulting in HJ-aggregates occurs.^7^

In the emission spectrum of the ribbons as seen in Figure 2B, we classify the 590 nm peak as the 0–0 electronic state transition, the 640 nm peak as the 0–1 vibronic transition, and the 700 nm shoulder as the 0–2 vibronic transition, based on the Franck–Condon principle.^47^ We conclude that the 590 nm peak is the 0–0 transition peak due to its strong temperature dependence, further explained later in this section. Similar to the absorption measurements, we plot the intensity of the 0–0 and 0–1 peaks of the J- and HJ-aggregates over varying concentrations, as shown in the inset of Figure 2B. By performing linear fittings, we determine that at increasing concentrations, the J-aggregate 0–0 peak increases at a rate of 17.4 kilocounts per micromolar, indicating an increase of molecules coupled together in aggregates due to forced proximity. Conversely, the 0–1 peak of the J-aggregates rises at a much lower rate of 0.611 kilocounts per micromolar, indicating that the molecules are forming large J-aggregate assemblies with limited vibronic coupling effects at higher concentrations. This trend is similar for the HJ-aggregates, though the 0–0 peak increased in intensity at a lesser rate of 6.03 kilocounts per micromolar. Additionally, the 0–1 HJ-aggregate peak increases at a very similar rate to the J-aggregates, at 0.582 kilocounts per micromolar. This suggests that for the HJ-aggregates, the interactions at higher concentrations are not purely J-like, or there is some mechanism that cancels out the J-like coupling. This leads us to the conclusion that there is H-like coupling in these ribbon aggregates. Much work has been done to characterize these newer weakly coupled aggregate structures. Researchers have recently designed models that take short- and long-range dipole coupling and charge transfer into consideration for the aggregates and have led to the classification of null, I-, and HJ-aggregates, which have features of both H- and J-aggregates.^6,21,22^ Due to the complex arrangement of molecules in 3D aggregates, they will not retain the ‘ideal’ classification of either H- or J-aggregates, as is the case in 1D chains. However, we can determine their classification based on their overall optical characteristics.^7,21^

One major indicator of a mixed aggregate type is through temperature-dependent emission intensities. The ratio between intensities, or *I* ^0–0^/*I* ^0–1^ = *R*_PL_, has been extensively studied in molecular aggregates and in terms of the ratio rule and can give an estimate of the exciton coherence number or the number of molecules that will cooperatively and coherently emit.^7,60^ The generalized ratio rule for disordered J-aggregates states that *R*_PL_ ≈ *N*_coh_/λ,^2^ where *N*_coh_ is the exciton coherence number, and λ^2^ is the Huang–Rhys factor (0.605 for PIC-Cl).^7,23,61^ It should be noted that the *R*_PL_ at 4 K varies greatly between our ribbons, as shown in the histogram in Figure S8. The varying *R*_PL_ between ribbons can be interpreted as the change in the number of coupled molecules or the exciton delocalization length in an aggregate structure. *R*_PL_ is known to depend on temperature as well and is shown to increase (decrease) in low temperatures for J-aggregates (H-aggregates).^7^ H-aggregates usually have a small *R*_PL_, and in the ideal case, approach zero. While the 0–0 transition peak is strongly dependent on temperature, due to it being a symmetric exciton state, the 0–1 transition peak does not change noticeably at varying temperatures.^7,20,21^ These effects are due to the lack of exciton–phonon coupling at low temperatures, which increases exciton delocalization in the aggregates and aggregate effects. For the J-aggregates measured at 4 K, as shown in Figure S8, the *R*_PL_ = 8.78 ± 4.04. This larger *R*_PL_ value that decreases to 5.22 ± 3.52 at room temperature can be used to classify the structure as a J-aggregate. For the HJ-aggregates, the *R*_PL_ = 1.60 ± 0.87, much smaller than that of the J-aggregates, but not approaching zero like H-aggregates. The ribbon HJ-aggregates *R*_PL_ further decreased to 0.58 ± 0.17 at room temperature or 295 K, behavior consistent with J-aggregates.^7^

While comparing the *R*_PL_ at 4 and 295 K initially indicates J-like coupling, a more detailed temperature-dependent study is necessary. The emission spectra at a series of temperatures are shown in Figure 2C. The intensity of the ratio of J-aggregates decreases with increasing temperature, an effect we previously described and attributed to the onset of electron–phonon coupling, causing the excitons to localize.^7^ However, for the PIC-I ribbons, the trend is not linear. We measured the temperature-dependent emission on four different samples and then calculated their *R*_PL_, as shown in Figure S9. These *R*_PL_ values were averaged and are displayed in the inset of Figure 2C. Notably, at 50 K, there is a sharp increase in the *R*_PL_ rising from 2.52 at 4 K to 3.17 at 50 K. After this sharp rise, the HJ-aggregate *R*_PL_ decreases to values below the initial 4 K *R*_PL_. This behavior is unexpected of H- and J-aggregates but has been characterized in HJ-aggregates.^7,20,21^ HJ-aggregates are a unique type of aggregate that host both H- and J-like coupling and are thermally activated. At low temperatures, the lowest exciton state is antisymmetric, resulting in H-aggregate-like behavior.^7^ As the temperature increases, the highest exciton state becomes thermally populated, allowing 0–0 transitions that were previously forbidden. This 0–0 emission intensity will peak at temperature *T*_P_, when the energy difference between the lowest antisymmetric and the highest symmetric exciton band is equal to *k*_B_ *T_p_*.^7,21^ After this temperature peak, the *R*_PL_ of HJ-aggregates will decrease in a nonlinear fashion due to the decreased thermal population of the symmetric 0–0 exciton band.^20–22,60^ While a thorough temperature dependence emission study is needed to determine the *T*_P_ of the PIC-I ribbons, the trend matched well with the described behavior of HJ-aggregates over multiple samples, leading us to conclude that the created ribbons were HJ-aggregates. As the research on weakly coupled aggregate types is still relatively new, we propose these previously undiscovered PIC-I ribbons as a novel method to easily study the HJ-aggregates and how cryoconcentration can be used to create new kinetically trapped aggregates.

To study the ordering of our aggregate structures, polarization-dependent PL measurements were performed. Polarized emission measurements were completed on two spots at 293 K, or room temperature, on the J- and HJ-aggregates, as displayed in Figure 3. As we cannot resolve individual J-aggregates with optical microscopy, a spot was chosen with a strong J-band or 0–0 transition peak emission, with the corresponding optical image shown in Figure 3B. The aggregates were excited with normal polarization (90°), and their polarized spectra were collected at varying polarization angles. Integrated intensities were extracted as described in the methods section and were plotted along their emission angle, shown in Figure 3C and D. The HJ-aggregate 640 nm or 0–1 transition peak displays a strongly polarized emission along the short axis of the ribbon. The 590 nm or 0–0 transition peak is also polarized along the short axis, but to a lesser extent. The J-aggregate showed strongly polarized anisotropy as well, which, according to prior reports, is along the long axis of the PIC-I J-aggregates.^7,9,49,62,63^ The molecules’ 0–0 transition is polarized along the TDM, making polarization anisotropy evidence for ordered aggregate structures in early reports. While it could be argued that the ribbons are completely disordered and are emitting due to AIE, the polarization of the 0–0 and 0–1 peaks indicates that the structures contain some ordered aggregate structure.^59^ Along with the integrated intensities, we calculated the *R*_PL_ of the ribbons as well. While the 0–1 transition peak is strongly polarized, the 0–0 transition peak is weakly polarized toward the short axis of the ribbon, which results in higher *R*_PL_ along the long axis of the ribbon. A polar plot of the *R*_PL_ as a function of emission angle is displayed in Figure S10. The smallest *R*_PL_ values were ∼0.35 at 90, 110, and 130°, where the measured 0–1 transition peak was its strongest, and approximately 90° away from the angle of the long axis of the ribbon. Conversely, at 30°, where the 0–1 transition peak was weakest and close to the angle of the long axis of the ribbon, the *R*_PL_ increased to 1.72, increasing by a factor of 4.91. By primarily collecting emission polarized along the long axis of a ribbon, experiments can be performed on the 0–0 transition peak while limiting the fluorescence contributions from the vibronic transitions. This polarized emission isolation could additionally be implemented to observe J-aggregate effects in complex aggregate structures that contain J- and H-aggregate coupling effects.

**Figure 3:**
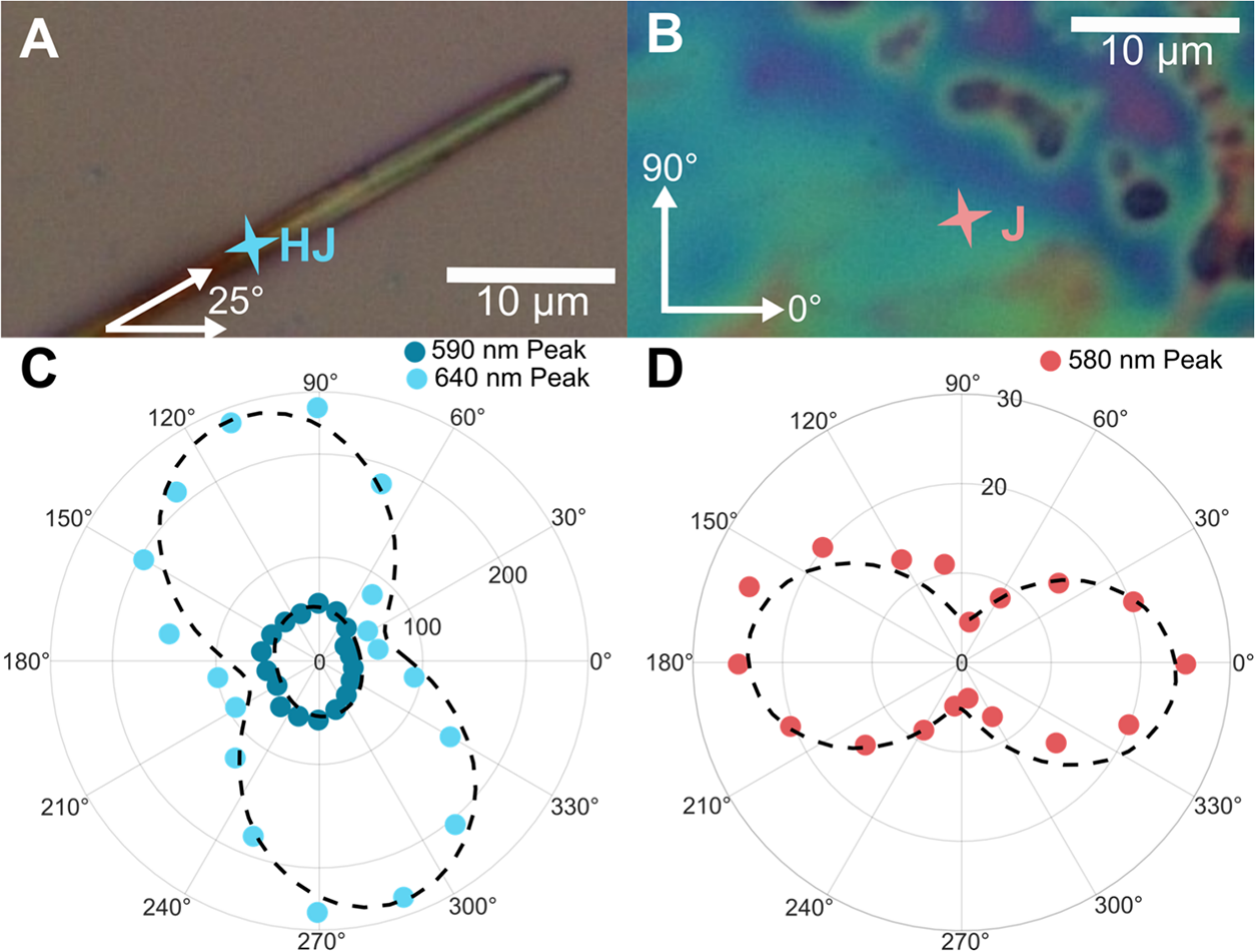
Polarized emission data of PIC-I aggregates. (A) Microscope image of HJ-aggregate. (B) Microscope image of J-aggregate. (C) Polar plot of integrated intensities of 590 and 640 nm peaks of HJ-aggregate emission. (D) Polar plot of 575 nm emission from the J-aggregate J-band.

While the steady-state spectral information on the ribbons explains their potential structure and alignment of TDMs, the time-resolved PL (TRPL) helps to further characterize their HJ-aggregate properties. The TRPL data of both J- and HJ-aggregates are displayed in Figure 4A, along with an iterative reconvolution of a single exponential with the IRF fitting. A fluorescence image of each of the spots measured is shown in the inset of Figure 4A. These measurements were performed at 4 K to initially observe the system without the effects of nonradiative decay pathways described earlier. The fitted lifetime value for the J-aggregate in Figure 4A is 132 ps, while the lifetime value for the HJ-aggregate is 702 ps. While PIC J-aggregates have been consistently studied since their discovery, their emission lifetimes will change depending on the concentration, laser intensity, laser wavelength, and their environment.^9,58,64–67^ Studies have reported the fluorescence lifetimes of PIC J-aggregates at room temperature as 400 ps,^27^ 310 ps in aqueous solution,^64^ and 710 ps in a thin liquid layer.^58^ Exciton–exciton annihilation (EEA) and photobleaching must also be taken into consideration as factors that could artificially shorten their lifetimes.^44,67^ To mitigate these factors, the samples were prepared at the same concentration, excited at the same laser fluence of 40 nW, and measured at 4 K. These results stayed consistent across different spots and samples.

**Figure 4:**
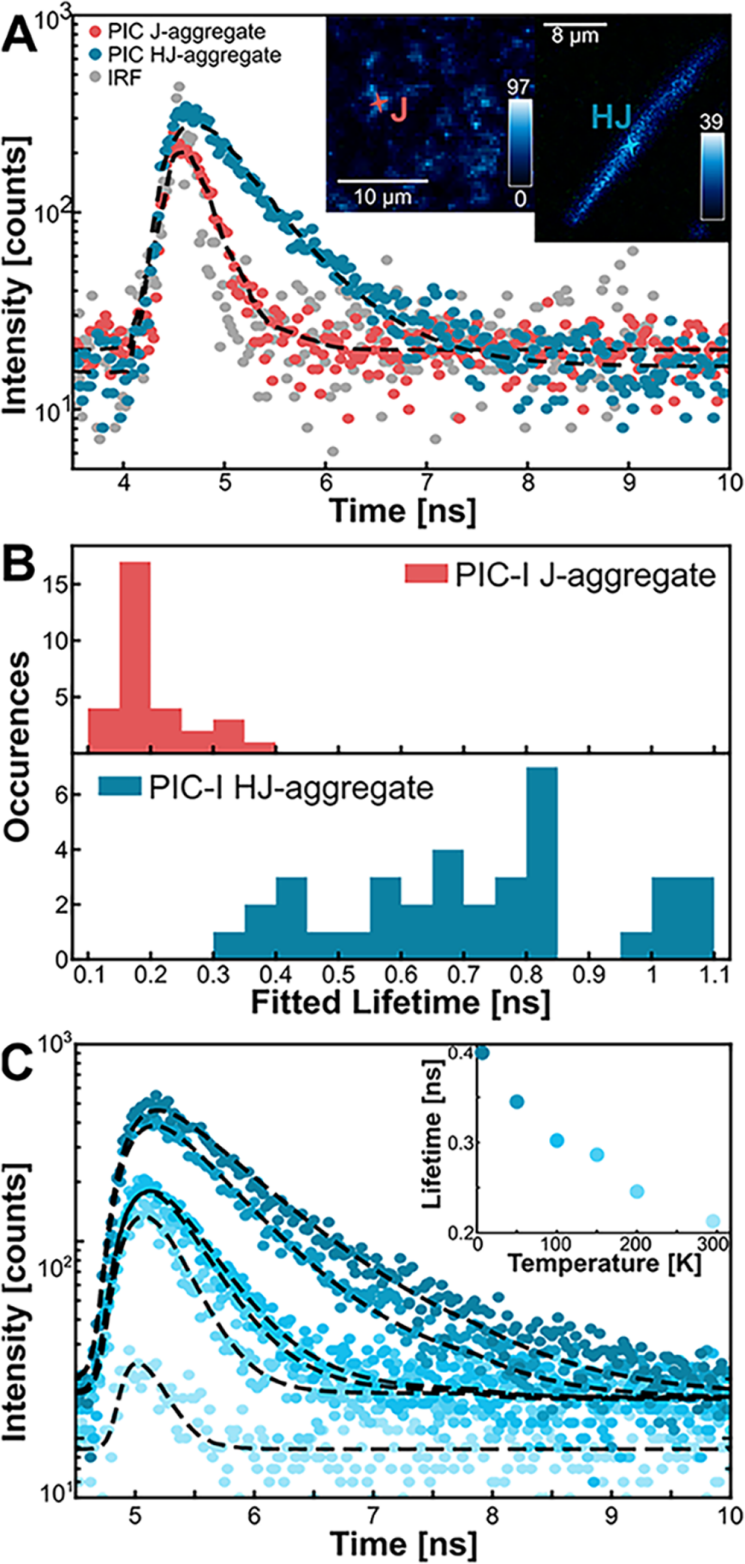
Fluorescence lifetime data of PIC-I aggregates. (A) IRF of the instrument (gray) and lifetime traces at 4 K for HJ-aggregates (blue) and J-aggregates (red), fitted with a single exponential convolved with the IRF (black dashed), with insets showing fluorescence images of the measured locations. (B) Histogram of fitted lifetimes for 33 J-aggregates (red) and 38 HJ-aggregates (blue) at 4 K. (C) Temperature-dependent lifetime measurements at a single location on an HJ-aggregate ribbon at 4, 50, 100, 150, 200, and 300 K.

As with the *R*_PL_ in the HJ-aggregate ribbons, the fitted lifetime values varied greatly between spots and samples, as shown in the histogram in Figure 4B. The J-aggregates’ average lifetime was 199 ± 58 ps, measured for 33 J-aggregate sample spots. This indicates consistent lifetimes for the J-aggregates, even when measured on different spots or samples. The HJ-aggregate sample, however, had an average and standard deviation lifetime of 718 ± 211 ps measured for 38 HJ-aggregates. This average is significantly larger than that of the J-aggregates, and their spread is much larger. This result is consistent with some reports of H-aggregates having a longer fluorescence lifetime compared to their monomer form, which HJ-aggregates will reflect at low temperatures.^68–70^ Similar to the varied *R*_PL_, the large spread in the HJ-aggregate lifetime results suggests that the exciton delocalization varies greatly across samples, due to disorder in molecule alignment or aggregate size. From these largely varied results, we can also hypothesize that the H-like coupling contributions are stronger in some aggregates, resulting in longer lifetimes.

The monomer fluorescence of PIC-I is very weak, with a reported quantum yield of 0.01–4% at room temperature.^41,71,72^ The radiative lifetime of PIC-I was calculated by Ermolaeva et al. as 10 ns using the Strickler–Berg formula and the absorption spectrum of PIC-I in methanol.^58^ This is the same environment where we measure the PIC-I monomer absorption and fluorescence, and we can generally estimate the fluorescence lifetime to be on the scale of 10s to 100s of ps.^58,64,66,67^ Without a notable monomer fluorescence, comparing properties in PIC-I is difficult, so we compared the fluorescence of our newly developed HJ-aggregate ribbons to their well-established J-aggregate form, which has been done previously.^67^

For the PIC-I J-aggregates in our studies, the average fluorescence fitted lifetime slightly decreased by 50 ps from 4 to 293 K, as displayed in Figures 4B and S11. A notable feature of collective emission in J-aggregates is the decrease in radiative decay lifetime as temperature decreases, which was previously observed in PIC-Br.^30^ As the temperature decreases, exciton–phonon coupling will not disturb the coherent coupling between the molecules as much, leading to greater exciton delocalization across the aggregate and increasing the effective number of emitters contributing to the collective emission. The number of collectively coupled molecules scales inversely to the radiative decay lifetime. This means J-aggregates fluorescent decay lifetime will decrease at low temperatures. The slight increase we observed for PIC-I J-aggregates’ fluorescence decay lifetimes does not indicate superradiance, but rather that there is not a strong dependence on the molecules’ nonradiative decay pathways. The quantum yield of thin liquid layers of PIC-I J-aggregates at room temperature has been previously reported to be 44%.^58^ Other researchers have estimated the quantum yield to be 28% in NaCl solution, which decreases to 3% in polymer thin films.^73^ The solid samples used here will have decreased quantum yield, due to the molecules becoming disordered in their aggregates after drying to thin films.^26^ Using the previously recorded radiative rate of 1.6 × 10^9^ per second for PIC-I J-aggregates,^58^ we can estimate the average quantum yield to be ∼25%.

The HJ-aggregates’ fluorescence lifetimes increased significantly with decreasing temperature, as seen in lifetimes in Figure 4C, increasing the mean and standard deviation from 175 ± 59 ps at 295 K for 33 samples to 718 ± 211 ps at 4 K for 38 samples. The fluorescence intensity trend is similar to that of the steady state PL, with the intensity at 50 K almost matching the 4 K intensity. The combination of the onset of J-like and exciton–phonon coupling at higher temperatures shortened the fluorescence decay lifetimes to values similar to those of the J-aggregates. As mentioned earlier, relative QY measurements revealed an approximate QY of 8% for the PIC-I ribbons at room temperature. We can then use this value to calculate the average radiative rate of 4.6 × 10^8^ per second for the HJ-aggregates. This estimated rate is lower than the recorded one for J-aggregates,^41,58,73^ which supports our claim for weakly coupled aggregates.

One key signature that researchers have used in the past to prove the presence of coupled emission in two-level systems is photon bunching in second-order autocorrelation measurements (g^(2)^(τ))^42,74–78^ by a Hanbury Brown-Twiss (HBT) interferometer.^78,79^ Bunching results from photons arriving at the detectors in ‘bunches.’ This super-Poisson distribution results in a peak in the second-order autocorrelation at time 0 ns, or *g* ^(2)^(0) > 1. Bunching is a feature of superradiance or collective emission events, but has not been observed in molecular aggregate systems larger than two molecules.^10,42^ This could be due to several reasons, primarily the fragile nature of the molecular aggregates, our inability to focus on a single cooperative aggregate, and EEA.^44,80^ Most J-aggregates cannot be isolated with visible light, making it difficult to focus only on the emission of a single cooperative aggregate. Another reason this measurement is hard to perform is the delicate nature of the aggregates. Held together only by intermolecular forces and susceptible to photobleaching, molecules in self-assembled aggregates can dissociate or be destroyed easily.^44^ Second-order autocorrelation measurements have been attempted previously by Stangl et al. on single J-aggregates of poly­(para-phenylene-ethynylene-butadiynylene) (PPEB), but they found that rather than bunching, they saw evidence of antibunching, which they conclude to be due to EEA.^80^

Due to the large *R*_PL_ and small fluorescence lifetimes at some spots on some ribbons, we presumed that J-aggregate-like contributions within the arrangement of the HJ-aggregates dominated. Pulsed second-order autocorrelation measurements were performed on the 0–0 transition peak at 4 K, where H-like coupling should dominate, over many different spots on various ribbons, displayed in Figure 5. While most spots resulted in a Poisson distribution or *g* ^(2)^(τ) ≈ 1, as shown in Figures 5D,E, a bunching effect was observed at some locations. Bunching is most notable for the first spot, its position shown in Figure 5A and its *g* ^(2)^(τ) response in Figure 5B. We find that we have *g* ^(2)^(0) = 1.22, a value greater than 1, and evidence of a super-Poisson distribution. Thermal and chaotic light sources also have a super-Poisson distribution.^79^ The molecular aggregate does not produce thermal light, as the power used here is 40 nW and the sample is kept at 4 K to prevent the onset of thermal emission after 50 K. Chaotic light is harder to distinguish, but we can rule out its contribution. Chaotic emitters will have second-order autocorrelation functions broadened with a Gaussian or Lorentzian line shapes and a *g* ^(2)^(0)=2.^79,81^ As our *g* ^(2)^ functions were not broadened and not equal to 2 at time 0, we can assert that the aggregates are not emitting chaotically. Additionally, our previous results provided evidence that the sample is an ordered and coupled HJ-aggregate, capable of delocalized states and radiative emission. Bunching due to chaotic emission is the statistical result of individual random emitters, a system inconsistent with our sample.^79^ A more likely source of this bunching is biexciton or multiexciton decay. We performed fluence-dependent measurements on the spot with the strongest bunching signal, spot 1, as seen in Figure S12, and found a linear trend, indicating that the bunching was not due to multiexciton decay. A possible source of this bunching feature is a cooperative emission process. Bunching was only found in some spots on the sample, while many other measured spots display a coherent Poisson distribution, seen in spots 3 and 4 in Figure 5D,E. We suspect that the differing emission characteristics coincide with a slightly different arrangement of the molecules due to their self-assembly. While J-aggregates are more likely to coherently and cooperatively emit, their g^2^ is overcome by high EEA rates, a rate that has been reported experimentally and theoretically to be lower for H-aggregates.^39,82–86^ The lowered rate of EEA for H-aggregates, along with suppressed nonradiative decay pathways, could explain why bunching was successfully observed for these weakly coupled type aggregates.

**Figure 5:**
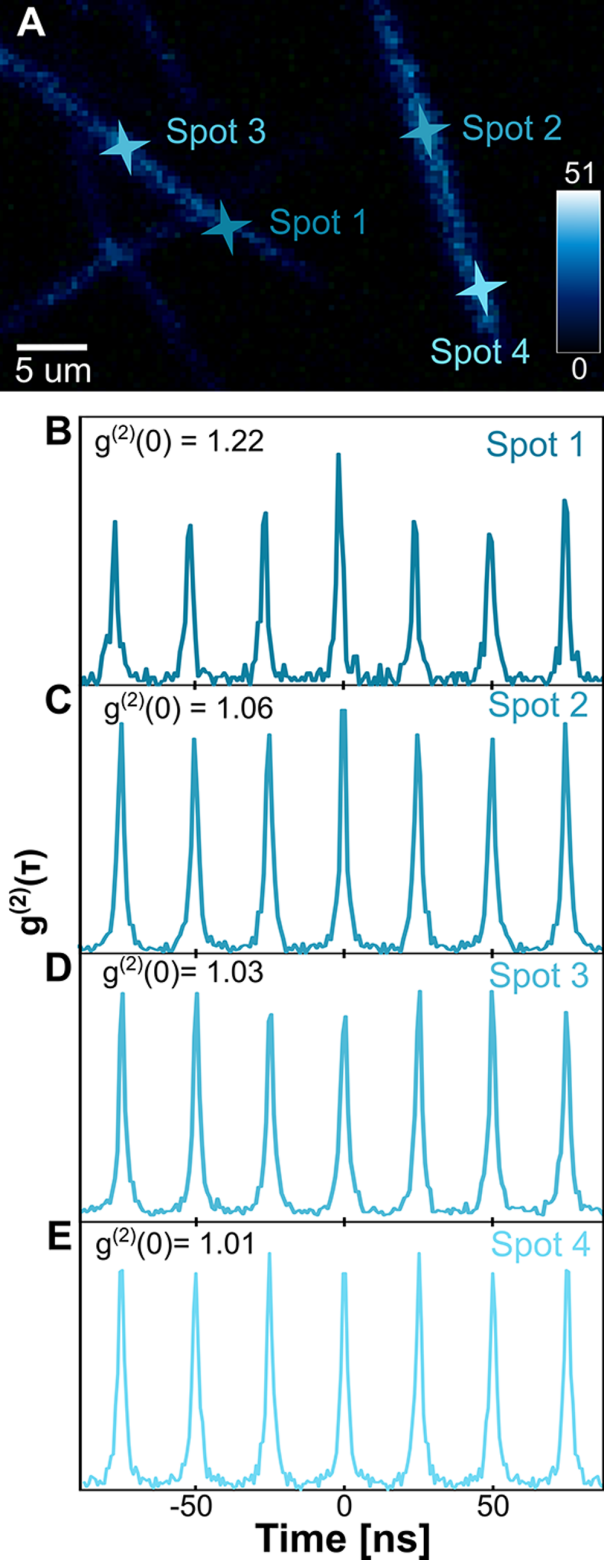
Second-order autocorrelation measurements on HJ-aggregate nanoribbons. (A) Fluorescence microscope image of nanoribbons labeled with spots 1, 2, 3, and 4. (B) Second-order autocorrelation of spot 1 showing photon bunching with *g* ^(2)^(0) = 1.22. (C) Spot 2 showing slight bunching with *g* ^(2)^(0) = 1.06. (D) Spot 3 showing Poissonian (coherent) emission with *g* ^(2)^(0) = 1.03. (E) Spot 4 showing Poissonian (coherent) emission with *g* ^(2)^(0) = 1.01.

## Conclusions

In this work, we developed and characterized emissive ribbon HJ-aggregates of PIC-I, which have not been previously studied. By developing a simple method to synthesize HJ-aggregates, we can isolate them from their readily available J-aggregate counterparts. A ribbon structure was formed of HJ-aggregates by embedding the dye in an aqueous medium, which was then frozen. The ribbon structures were unique compared to the 2–3 nm width fibers that PIC-I J-aggregates form.^23,36^ These PIC-I HJ-aggregates assumed a variety of sizes and could achieve lengths over 1 mm. We characterized their spectral absorption and emission, discovering a similar absorption line shape to that of the monomer, as well as three red-shifted emission peaks compared to the PIC-I monomer form. These results, as well as temperature-dependent *R*_PL_, indicated that mixed coupling existed in the aggregate structure. We found polarized emission from ribbons along their short axis, the opposite case for J-aggregate fibers, suggesting an organized arrangement of molecules. The changed *R*_PL_ of the ribbons due to their polarization suggested a potential method to isolate the 0–0 electronic transition, as well. The fluorescence decay lifetimes at 4 K of the HJ-aggregates were revealed to be on average 3.6 times larger than those of the J-aggregates, while they maintained similar fluorescence decay lifetimes at 295 K, near ∼150 ps, once again displaying temperature-dependent hallmarks of HJ-aggregates. We also found that bunching occurred at some locations, which has not been reported for molecular aggregates larger than two molecules.^42^ This bunching is indicative of cooperative emission that could arise from varied orientation in some ribbons. While prior works only studied PIC J- and H-aggregates, our work, by developing a simple approach to synthesize PIC HJ-aggregates, fills the gap in studying new complex molecular aggregates. These HJ-aggregates, showing unique optical properties, will be a valuable addition to the reservoir of optoelectronic materials and could lead to the development of designer optoelectronic devices in the future.

## Supplementary Material



## Notes

Computational image analysis of PIC-I HJ-aggregate ribbons with histogram data of width, length, and aspect ratio; optical microscope image of a large PIC-I HJ-aggregate ribbon; additional SEM images of PIC-I HJ-aggregate ribbons; microscope images of HJ-aggregate solution drop-cast at various temperatures; time series of absorption of PIC-I HJ-aggregate ribbons in solution; relative QY measurements for HJ-aggregates; *R*_PL_ of HJ and J-aggregates of PIC-I at 295 K (room temperature) and 4 K; temperature dependence of *R*_PL_ of individual HJ-aggregates; polarization dependence of *R*_PL_ of HJ-aggregate ribbon; histogram of fluorescence decay lifetimes of PIC-I J- and HJ-aggregates at 295 K (room temperature); fluence dependence of integrated intensity of HJ-aggregate ribbons (PDF)
