# Supplementary material for: Discovery of Crystallized and Weakly Coupled Aggregates of Pseudocyanine Iodide
Source: J Phys Chem B. 2026 Jan 21;130(5):1622–33. doi: 10.1021/acs.jpcb.5c06896 (PMC12884520; doi:10.1021/acs.jpcb.5c06896)
Supplement: Supplementary file 1 [file jp5c06896_si_001.pdf]

Discovery of Crystallized and Weakly Coupled Aggregates of Pseudocyanine Iodide

Supplemental Information

Autumn Bruncz[1], Arka Chatterjee[1], Henry Gatica-Gutierrez[2], Sadie Brasel[1], Alexey Belyanin[3], Anna-Karin Gustavsson[1,2,4,5,6,7], Shengxi Huang[1]*

[1]Department of Electrical and Computer Engineering, Rice University, Houston, TX 77005, USA

[2]Department of Chemistry, Rice University, Houston, TX 77005, USA

[3]Department of Physics & Astronomy, Texas A&M University, College Station, TX 77840, USA

[4]Department of BioSciences, Rice University, Houston, TX, 77005 USA

[5]Smalley-Curl Institute, Rice University, Houston, TX, 77005 USA

[6]Center for Nanoscale Imaging Sciences, Rice University, Houston, TX, 77005, USA

[7]Department of Cancer Biology, University of Texas MD Anderson Cancer Center, Houston, TX, 77005 USA

*Corresponding Author, email: shengxi.huang@rice.edu, phone: 713-348-0000

Table of Contents

Figure S1: Image analysis figures ……………………………………………………………………..……………S3

Figure S2: Histogram data of the sizes of nanoribbons…………………………….………..…………S3

Image analysis details…………………………………………………………………………………S4

Figure S3: Optical image of the largest HJ-aggregate ribbon sample……………………………….……S4

Figure S4: Additional SEM images…………………………………………………………….……………S5

Figure S5: Images of HJ-aggregate ribbons deposited at varying solution temperatures………………..S6

Figure S6: HJ-aggregate dissociation in liquid over time………………………………………………..S7

Figure S7: Relative QY data and calculations for HJ-Aggregates……………………………..………S8

Figure S8: Histogram of R_{PL} factor for HJ- and J-aggregates (room temperature and 4K)……….….….S9

Figure S9: Temperature-dependent R_{PL} individual HJ-aggregate ribbons……………………….…..S9

Figure S10: Polarized dependence of R_{PL} of HJ-aggregates…………………………………….…………S10

Figure S11: Fluorescent lifetime histogram of HJ- and J-aggregates at room temperature……….…..S11

Figure S12: Fluence dependence of HJ-aggregate sample spot 1 that has bunching…………………..S11

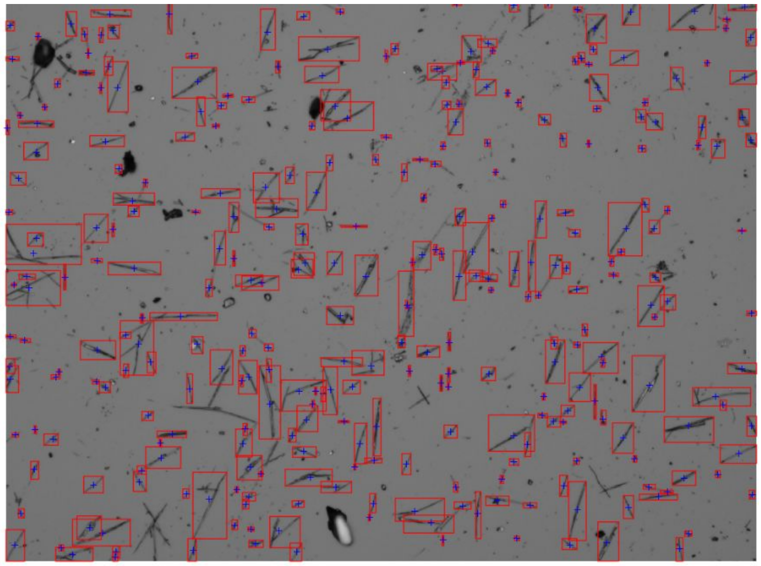

Figure S1: Microscope image of HJ-aggregate ribbons drop-cast onto a SiO_2 substrate and coated with a sucrose/trehalose mixture. Red boxes are shown around identified nanoribbon structures

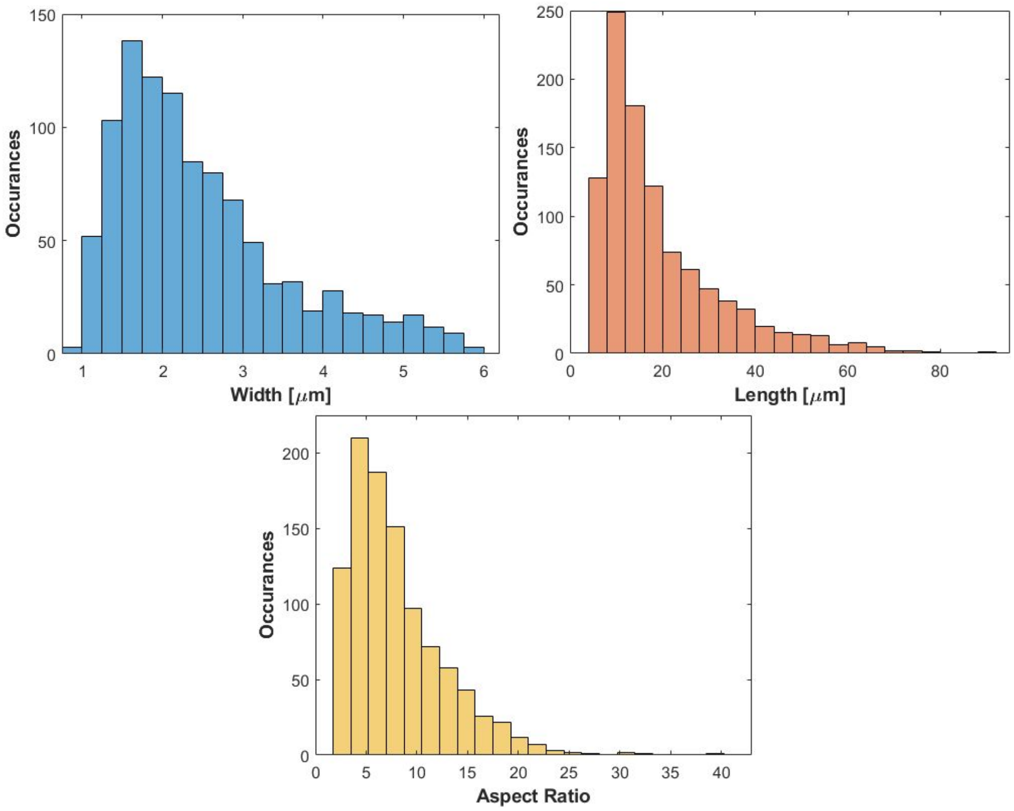

Figure S2: Histogram data of the 1024 ribbons collected with their width in blue, length in orange, and their aspect ratio in yellow. The average width, length, and aspect ratio were 2.5±1.1 μm, 19.3±13.3 μm, and 8.1±2.8, respectively.

Size analysis from microscope images: Images taken were uploaded to MATLAB, converted to greyscale, and then binarized to separate the nanoribbons from their background. The bwareaopen operation was used to remove artifacts of small, isolated pixels that didn't correspond to aggregates. The varying intensities of the nanoribbons in the optical microscope images resulted in island structures inside them once the images were binarized, which was fixed by using the imfill operation to fill enclosed objects. Once a clean binary image was produced, the regionprops function was used to calculate the geometric properties of all of the labeled objects. Here we collected the nanoribbons' short axis, long axis, and their aspect ratio. Any nanoribbon with a width larger than 5 μm or an aspect ratio less than 2 was identified in the image to ensure that they were single nanoribbons. Any identified object that was not a single nanoribbon was filtered out from the final calculations. All data were exported, and statistical analysis was performed on all 1024 identified nanoribbons. This method allows us to extract large quantities of data from nanoribbons quickly, but there is the possibility of some small error from our filtering process. With the overwhelming number of nanoribbons we were able to identify, any incorrect data will most likely be averaged out.

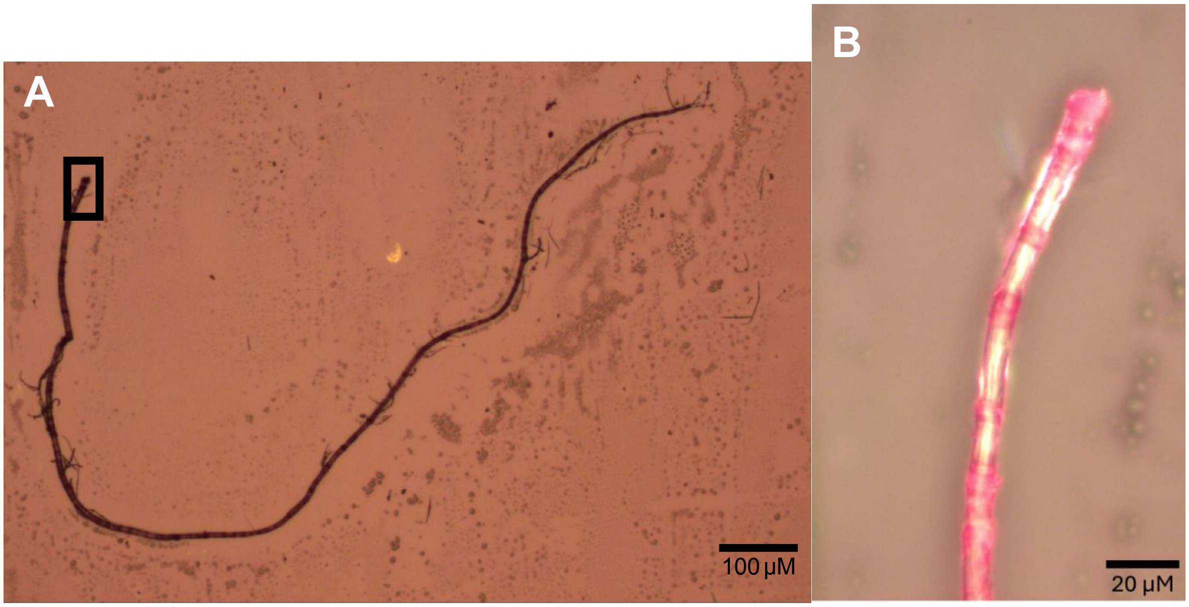

Figure S3: Optical microscope images of the large HJ-aggregate ribbon. The width is ~10 μm and its continuous length is ~1.1 mm. (A) image of the entire ribbon structure and (B) a close-up of the end of the ribbon in the black box in A.

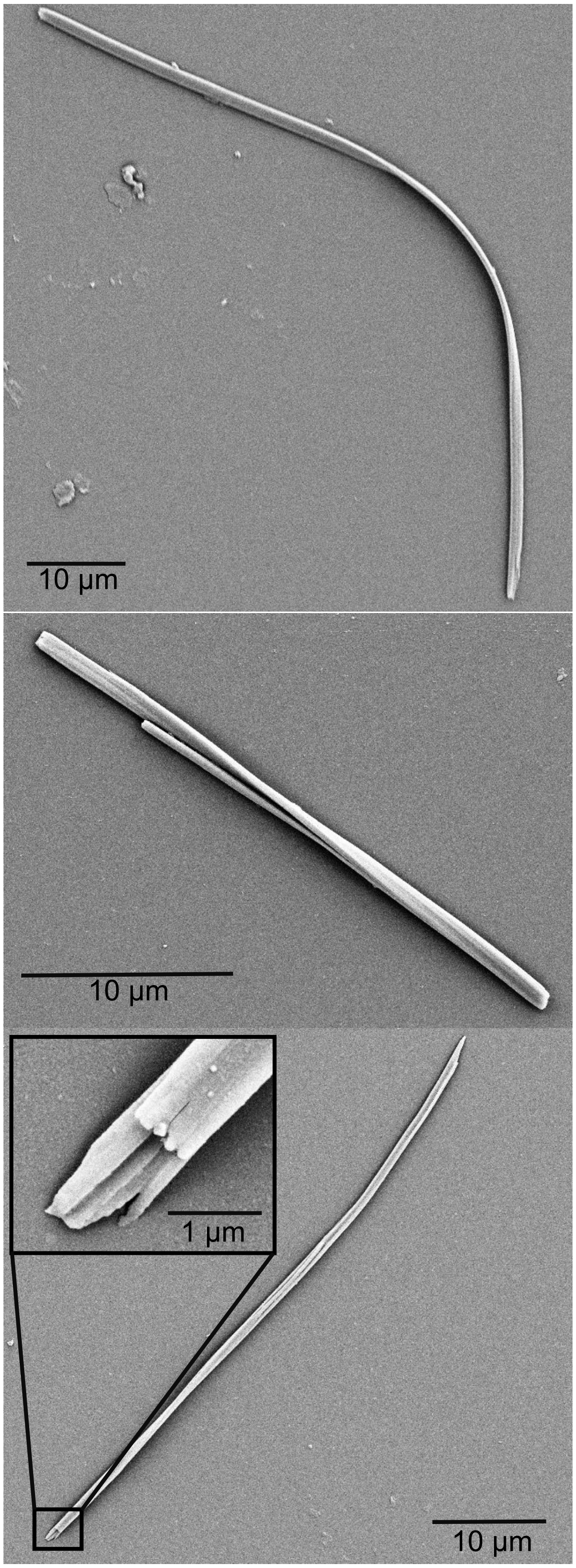

Figure S4: SEM images of various HJ-aggregates showing their nanoribbon structure.

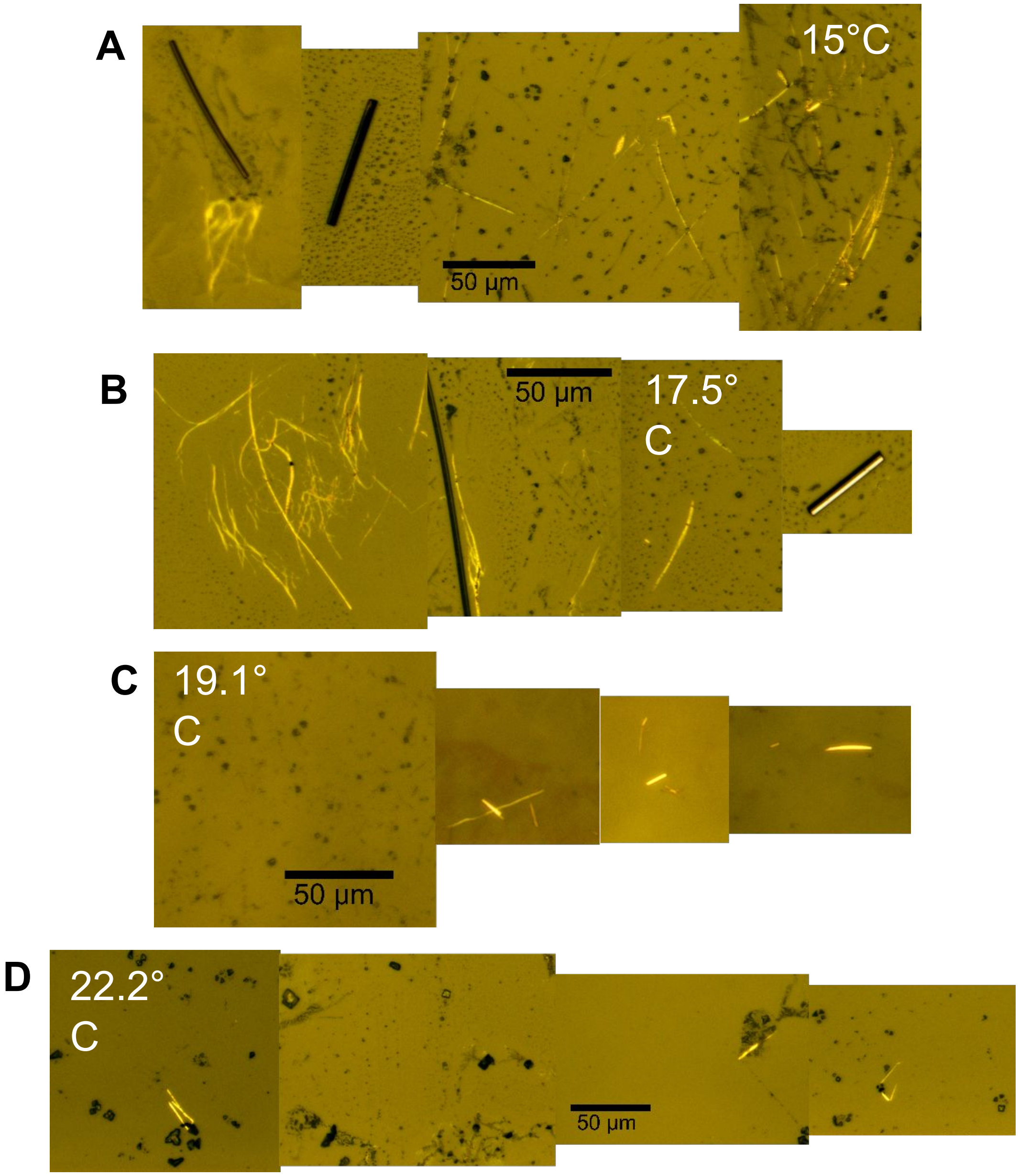

Figure S5: Optical microscope images of the HJ-aggregates after being deposited from a solution at varying temperatures: 15°C (A), 17.5 (B), 19.1 (C), and 22.2 (D). Images show characteristic dispersions of ribbons seen in yellow and black.

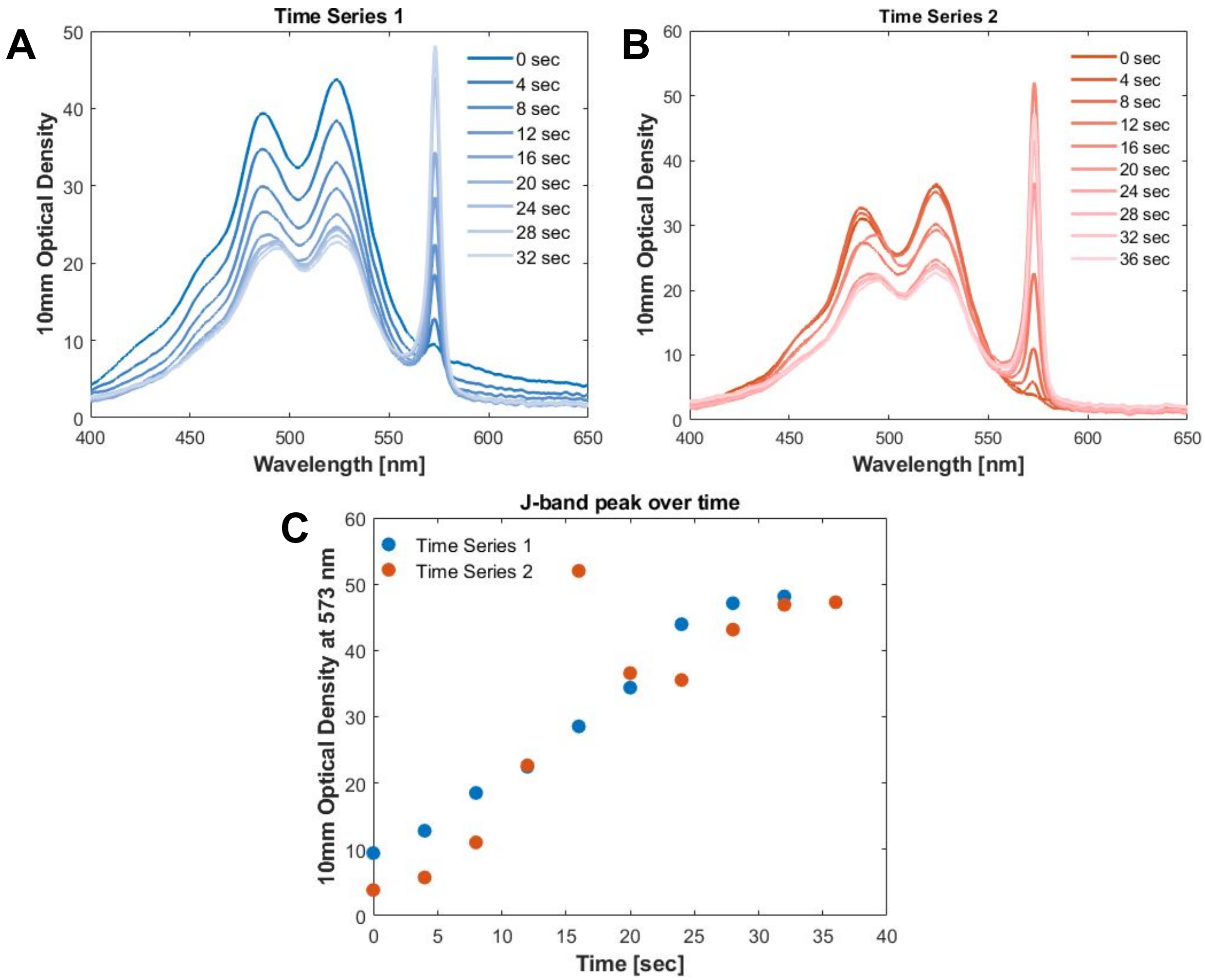

Figure S6: HJ-aggregate dissociation to J-aggregates in liquid over time through absorption measurements. (A) and (B) shows the rise in J-band at 573 nm in liquid solution in 4-second increments immediately after the sample is deposited from its frozen solution, (c) shows the peak height at 573 nm as a function of time, rising before plateauing at ~30 seconds for two separate samples.

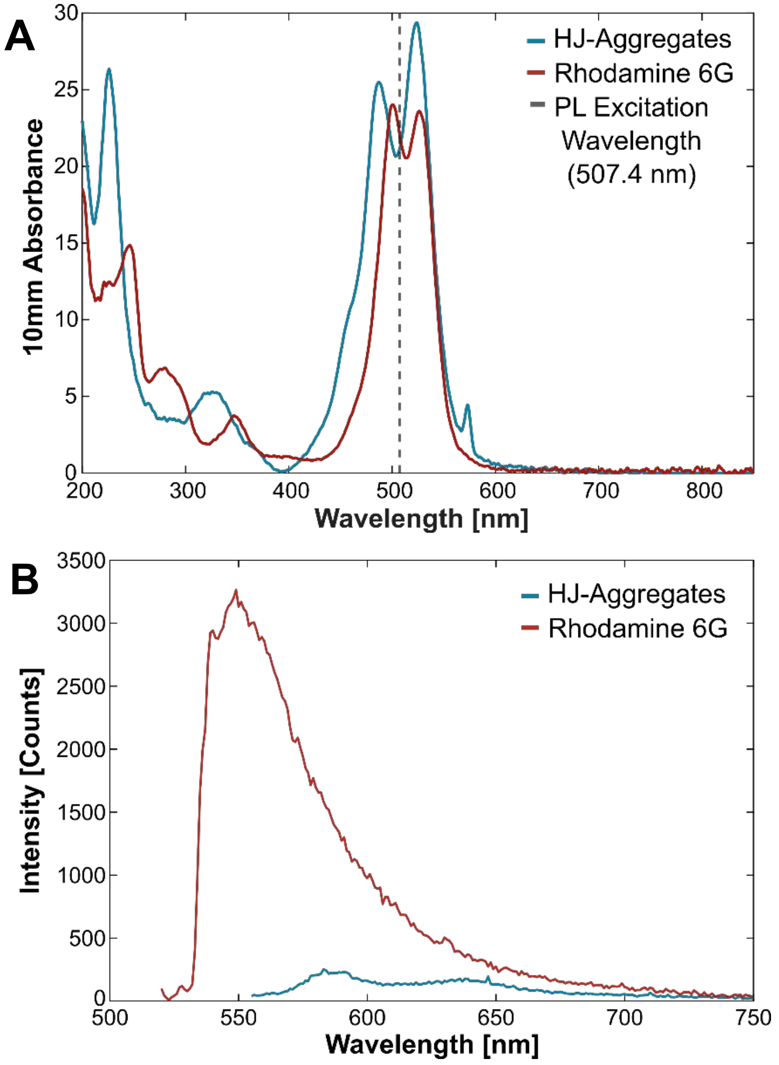

Figure S7: Experimental details of relative QY calculations. QY was determined using the following equation: $\Phi = \Phi_R \times \frac{Int}{Int_R} \times \frac{1-10^{A_R}}{1-10^{A}} \times \frac{n^2}{n_R^2}$ where Φ is the QY, Int is the integrated intensity, A is the absorbance, and n is the refractive index (1.3354 for 0.2 M NaCl aqueous solution[1]). The subscript R denotes the relative or known dye properties, for which we used rhodamine 6G (R6G) in water which has a recorded quantum yield of 82%.[2] By using 1 mM of the HJ-aggregate solutions and 0.188 mM of R6G, we were able to essentially cancel out the absorption term, as it has the same absorbance value at our PL laser excitation wavelength. PL measurements were recorded on thin liquid layers of the R6G and HJ-aggregates on glass coverslips with the PMA-Hybrid 50 detector as described in the methods section of the paper. PL was measured with 5x objective at 5 random locations on the sample, as to replicate bulk PL. As this method considers one concentration and utilizes microscopic PL, it is only an estimate of the QY in comparison of R6G. Further work and specialized equipment is needed to calculate the absolute quantum yield of the HJ-aggregates.

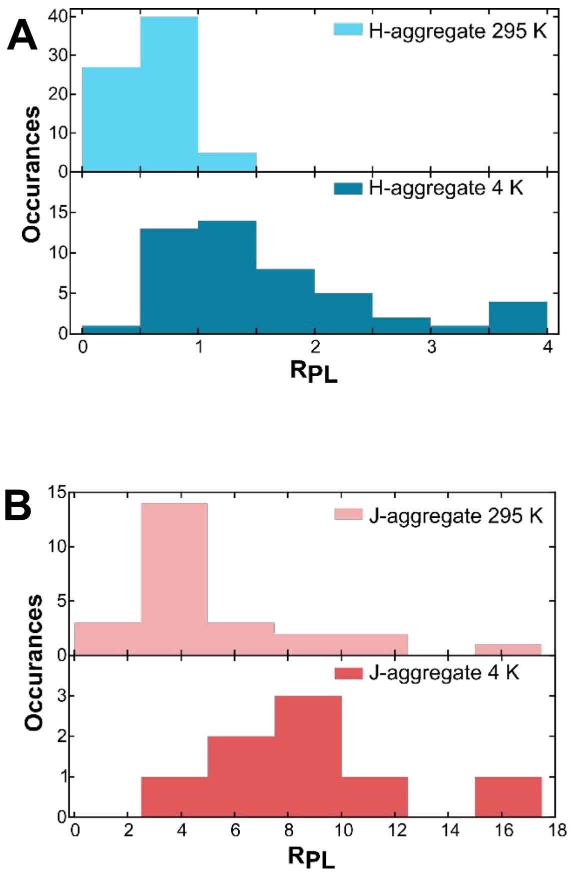

Figure S8: The R_{PL} of HJ-aggregates (A) and J-aggregates (B) at 295 K or room temperature (light value) and 4 K (dark value. The HJ-aggregate R_{PL} mean and standard deviation at 295 K and 4 K was 0.58±0.17 and 1.60±0.87, respectively. The J-aggregate R_{PL} mean and standard deviation at 295 K and 4 K was 5.22±3.52 and 8.78±4.04, respectively.

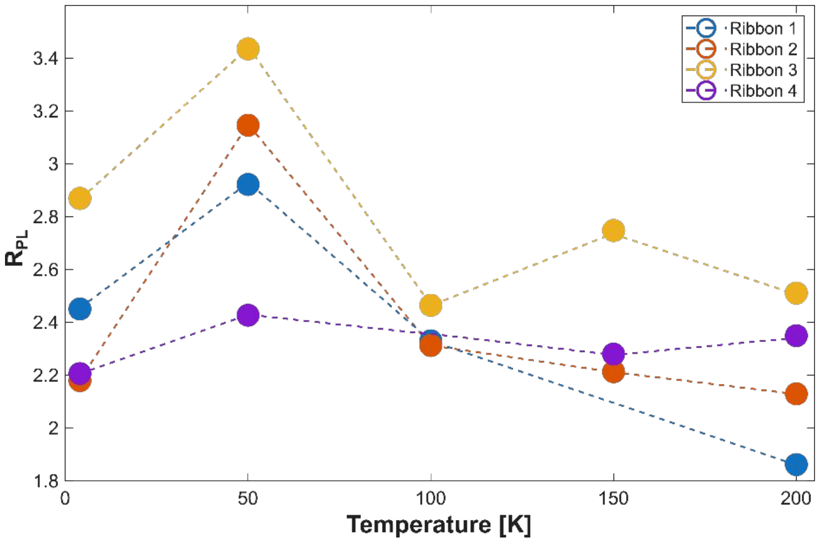

Figure S9: Temperature-dependent R_{PL} over four different HJ-aggregate ribbon samples. Averaged values are displayed in inset of main text Figure 2C.

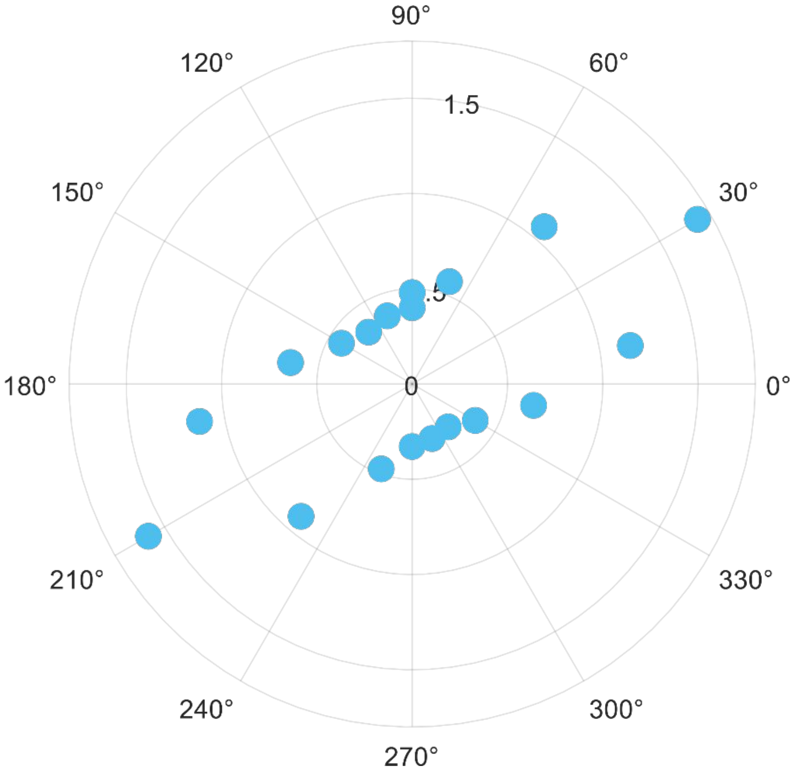

Figure S10: The polarized R_{PL} of HJ-aggregates. The optical image of the ribbon used in these experiments is in the main text as Figure 3A. The R_{PL} shows a higher R_{PL} value along the long axis of the ribbon.

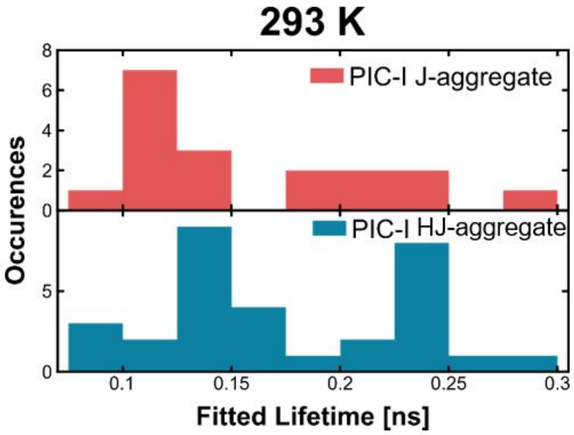

Figure S11: Histogram of lifetimes of J- and HJ-aggregates at 295 K or room temperature. The mean room temperature lifetime values for J- and H-aggregates were 156 ps and 175 ps, respectively. The standard deviations of the room temperature lifetime values for J- and H-aggregates were 55 ps and 59 ps, respectively.

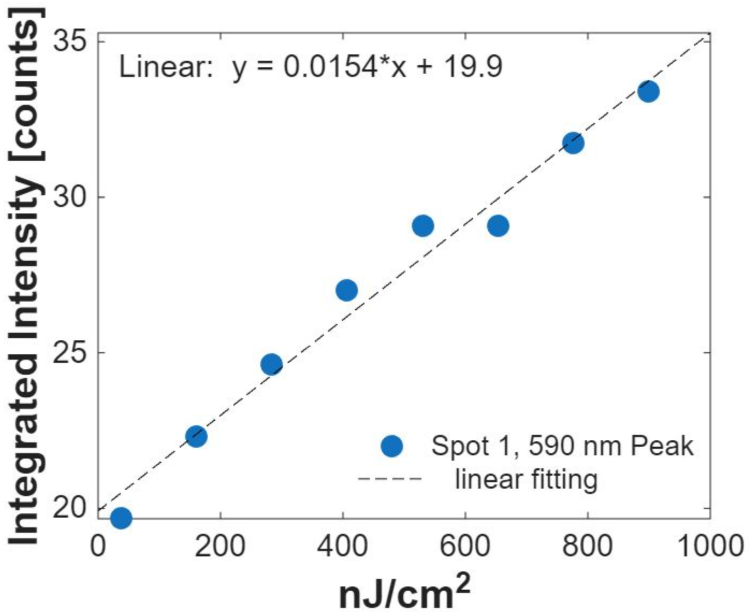

Figure S12: Fluence dependence of integrated intensity of the time-resolved single photon counting over a 30-second period centered on 590 nm with a 5 nm band pass focused on spot 1 of Figure 5. All spots were measured in random order.
